# Development of an Immunosensor for *Pf*HRP 2 as a Biomarker for Malaria Detection

**DOI:** 10.3390/bios7030028

**Published:** 2017-07-18

**Authors:** Aver Hemben, Jon Ashley, Ibtisam E. Tothill

**Affiliations:** 1 Surface Engineering and Nanotechnology Institute, Cranfield University, Cranfield, Bedfordshire MK43 0AL, UK; a.hemben@cranfield.ac.uk; 2 Department of Micro- and Nanotechnology, Technical University of Denmark, Produktionstorvet, 2800 Kgs. Lyngby, Denmark; jash@nanotech.dtu.dk

**Keywords:** Malaria, *Pf*HRP 2, parasites, immunosensor, biosensor, nanoparticles

## Abstract

*Plasmodium falciparum* histidine-rich protein 2 (*Pf*HRP 2) was selected in this work as the biomarker for the detection and diagnosis of malaria. An enzyme-linked immunosorbent assay (ELISA) was first developed to evaluate the immunoreagent’s suitability for the sensor’s development. A gold-based sensor with an integrated counter and an Ag/AgCl reference electrode was first selected and characterised and then used to develop the immunosensor for *Pf*HRP 2, which enables a low cost, easy to use, and sensitive biosensor for malaria diagnosis. The sensor was applied to immobilise the anti-*Pf*HRP 2 monoclonal antibody as the capture receptor. A sandwich ELISA assay format was constructed using horseradish peroxidase (HRP) as the enzyme label, and the electrochemical signal was generated using a 3, 3′, 5, 5′tetramethyl-benzidine dihydrochloride (TMB)/H_2_O_2_ system. The performance of the assay and the sensor were optimised and characterised, achieving a *P*fHRP 2 limit of detection (LOD) of 2.14 ng·mL^−1^ in buffer samples and 2.95 ng∙mL^−1^ in 100% spiked serum samples. The assay signal was then amplified using gold nanoparticles conjugated detection antibody-enzyme and a detection limit of 36 pg∙mL^−1^ was achieved in buffer samples and 40 pg∙mL^−1^ in serum samples. This sensor format is ideal for malaria detection and on-site analysis as a point-of-care device (POC) in resource-limited settings where the implementation of malaria diagnostics is essential in control and elimination efforts.

## 1. Introduction

Malaria is a serious disease that is caused by an Apicomplexan Plasmodium parasite that is transmitted by adult female Anopheles mosquitoes, which thrive in tropical and subtropical weather [1]. Malaria affects approximately 50% of the world’s population, and causes millions of deaths [2]. According to the latest World Health Organisation (WHO), estimates, released in December 2016, there were 212 million cases of malaria in 2015, and 429,000 deaths [2]. From this, the African region accounted for the most global cases of malaria (88%), followed by the South-East Asia Region (10%) and the Eastern Mediterranean Region (2%). Despite control efforts, the disease continues to affect productivity, and therefore an effective diagnosis is required for the successful treatment and reduction of both complications and mortality [2].

The methods available for the detection of malaria include blood film microscopy, immune-chromatographic tests, and serological tests. Blood film microscopy shows the highest specificity, as it depends on the detection of *Plasmodium* parasites in blood circulation, and in some cases is essential for epidemiological purposes [3]. This assay is known as the gold standard method for malaria diagnosis despite problems with its field accuracy, unacceptably high false-positive rates, errors in species identification, and its operator-dependence [4,5]. Alternative methods, such as laser desorption mass spectroscopy (LDMS), loop mediated isothermal amplification (LAMP), and flow cytometry (FCM) are expensive, time consuming, require specialised training, and are characterised by various levels of sensitivity or specificity in relation to sample quality [6,7,8]. Levels of parasitemia are not necessarily correlative with the progression of the disease, particularly when the parasite is able to adhere to blood vessel walls. Therefore, more sensitive, easy to use diagnostic tools need to be developed in order to detect low levels of parasitemia in the field [9].

Serological malaria tests are blood tests that picks up the specific malaria antibodies produced by the immune system [10]. These methods have a specific use, as they are limited to the measurement of past exposure to the disease. Methods based on parasite nucleic acid detection [11] have shown great sensitivity and specificity, but require significant infrastructure and training, and are more expensive than the blood smear method [12]. Methods based on the use of antibodies to recognise parasite components or biomarkers have also emerged in recent years [13].

*Plasmodium falciparum* histidine-rich protein 2 (*Pf*HRP 2) is a 35 kDa protein comprising unique tandem repeats (Ala-His-His-Ala-Ala-Asp), and is present in the serum of a malaria-infected patient as a parasite antigen [14,15]. *Pf*HRP 2 is also present in food vacuole [16], digestive vacuole [17], and the membrane surface of the infected red blood cells [18]. *Pf*HRP 2 is produced in large amounts by the most lethal of malaria parasites, and is specific to *Plasmodium falciparum*. Other malaria biomarkers, such as parasite lactate dehydrogenase (pLDH), and/or parasite aldolase, are common to all Plasmodium species [19]. In addition, *Pf*HRP2 has been proven to be useful in detecting the presence of parasites in cases of placental malaria [20]. The significance of *Pf*HRP 2 has led to a lateral flow dipstick test [21,22], enzyme-linked immunosorbent assay (ELISA) tests [4], and Western blotting [23] for the clinical diagnosis of malaria in support of microscopy. The drawbacks of these techniques are that they are either of low sensitivity, which could lead to inappropriately withholding treatment from patients with malaria [19], or are as time consuming as the lab-based methods. Recent literature has reported the development of different sensors for malaria based on biomarker and antibody detection [24,25]. Therefore, in this work, we investigated the development of a rapid and highly sensitive sensor based on a screen-printed device, which would enable its use in low-resource countries. The method selected was based on chronoamperometry, which is well known for its high sensitivity [26] and ability to amplify a signal using nanotechnology. The use of biomarker detection related to parasite infection was also implemented in this work through the selection of *Pf*HRP 2. The biosensor was then optimised to achieve a sensitive outcome and a capacity to work in resource-limited settings, and can be combined with other biomarkers for malaria infection detection.

## 2. Materials and Methods

### 2.1. Materials

*Plasmodium falciparum* histidine-rich protein 2 recombinant protein (PIP001), sandwich pair HuCAL capture monoclonal antibody (HCA 160, IgG1, clone 14971), and detection (HCA 159, IgG1, clone 14964) monoclonal antibody conjugated to horseradish peroxidase (HRP) were purchased from AbDSerotec (UK). Phosphate buffered saline tablets (PBS, pH 7.4), PBST (0.05 *v*/*v* Tween-20), Tween-20, microtitre plates, and MaxiSorp (Nunc Immuno), were purchased from Thermo Fischer Scientific (Hertfordshire, UK). Bovine serum albumin (BSA), phosphate citrate buffer tablets, sodium hydroxide, potassium chloride (KCl), sodium carbonate, sodium bicarbonate, 3, 3′, 5, 5′-tetramethyl benzidine hydrochloride hydrate (TMB) (powder), colloidal gold, hydrogen peroxide, 95% ethanol, potassium ferricyanide [K_3_Fe(CN)_6_], and human serum were purchased from Sigma-Aldrich (Dorset, UK). Milk concentrate blocking solution was purchased from KPL (Gaithersburg, MD, USA). Double-distilled ultrapure water produced by a Millipore Direct-Q^®^ 3 UV (Millipore; Molsheim, France) was used for the analysis. All of the chemicals and solvents were of analytical or HPLC grade, and were used without further purification.

### 2.2. Sensors Fabrication and Electrochemical Measurements

Screen-printed gold electrodes (SPGE), consisting of a gold working electrode, a carbon counter and a silver–silver chloride pseudo-reference electrode were fabricated using a procedure similar to that described by Noh and Tothill [27], and printed using the facilities at DuPont with inks provided by the company (DuPont Microcircuit Materials, Bristol, UK). Three electrode batches, JD1, JD2a, and JD2b, were tested. The printing pastes used for JD1 were BQ221 carbon, BQ331 gold, 5880 Ag/AgCl, and 5036 blue encapsulant (DuPont Ltd. Bristol, UK), produced in 2010. JD2a and JD2b were different from JD1 in that the carbon ink used was BQ226, but all other inks used were the same as JD1. The JD2a sensors were from a batch produced in 2013, while the JD2b sensors were freshly produced (2015). The gold working electrode had a 5 mm diameter, giving a 19.6 mm^2^ planar area, and was printed on a graphite ink layer (dried at 120 °C, 30 min).

The electrochemical procedures were conducted using a computer-controlled four channel Autolab electrochemical analyser multipotentiostat (Metrohm, The Netherlands) throughout, which allows the simultaneous detection of four sensors. Data capture was through the supplied GPES version 4.9) software installed onto a personal computer (PC). The sensor edge connectors were from PalmSens (Provided by Alvatek, Gloucestershire, UK). The electrodes were characterised using cyclic voltammetry (CV) and chronoamprometry. The CV scans were conducted by using a 100 µL drop of potassium ferricyanide (K_4_Fe(CN)_6_3H_2_O) at 0.1, 0.5, and 1 mM in 0.1 M KCl, placed onto the electrode’s surface. Three scans were taken at varying scan rates (10, 20, 50, 70, and 100 mV·s^−1^) relative to the on board Ag/AgCl reference electrodes. The active area of the working electrode was calculated [28] using the Randles–Sevcik equation [29].

For sample analysis, each of the measurements was carried out in triplicate using a new strip in a non-deaerated and unstirred solution. For the selection of the optimal constant potential for the enzymatic reaction (TMB-H_2_O_2_-HRP), choroamperometry was conducted using a bare screen-printed gold electrode with buffer solution (50 mM phosphate citrate buffer, pH 5.0, in 0.1 M KCl) and a substrate (4 mM TMB, 0.06% H_2_O_2_) with an antibody-HRP conjugate. Step amperometry was conducted at a range of potentials from +600 mV to −400 mV within 600 s for the TMB-H_2_O_2_-HRP system, in order to achieve the best signal-to-noise ratio. Data plotted from the steady state current was used to obtain the concentration of the analyte. Following the measurements, the data were copied to Microsoft Excel for representation.

### 2.3. SEM and AFM Scan of the SPGE

Scanning electron microscope (SEM) (Phillips, Guildford, UK) was used to visualise the surface structure of the gold working electrode at 50× and 3500× magnification. An electron emission spectrum was also obtained by using the Environmental Scanning Electron Microscope (ESEM) to determine the composition of the SPGE. Atomic force microscopy (AFM) (Digital instruments, Boston, MA, USA) was used to obtain the sensor’s surface topography at 25 and 50 mu magnification.

### 2.4. Immunoassay Development (ELISA)

ELISA tests were first developed using micro well polystyrene plates, MaxiSorp (Nunc Immuno). A direct assay was first developed by adapting the standard ELISA AbDSerotec protocol (Abdserotec.com). Following optimisation, *Pf*HRP 2 recombinant protein (PIP001) was serially diluted in sodium bicarbonate buffer (pH 9.6) to yield concentrations of 0.01, 0.1, 0.5, 1.0, 5.0, 10, and 100 µg∙mL^−1^, then 100 µL of the antigen solution was added in triplicate to the plate and incubated at 4 °C overnight. The control wells contained no antigen. The plate was aspirated and washed three times using 200 µL 0.01 M PBS Tween-20 (0.05 *v*/*v*). A 200 µL of 1% BSA was then used to block the plate by incubating at 37 °C for 2 h in a Labsystems iEMS Incubator/shaker (Bradenton, FL, USA.). The wash steps were repeated. A detection antibody conjugated with horseradish peroxidase (HCA 159, 10 µg∙mL^−1^) was added to the wells, and incubated at 37 °C for 2 h. The plate was washed three times in PBS Tween-20 (0.05 *v*/*v*). A 100 µL solution of 3, 3′, 5, 5′ Tetramethylbenzidine/H_2_O_2_ was added to the reaction wells, and incubated at room temperature for 15 min in the dark. The reaction was stopped using 50 µL of 1 M H_2_SO_4_, and read at 450 nm on a Varioskan plate reader (Thermo Fischer Scientific (Hertfordshire, UK).

A Sandwich ELISA assay was then developed, where 100 µL of *Pf*HRP 2 capture antibody (HCA 160) was dissolved in 900 µL of 0.1 M sodium bicarbonate buffer (pH 9.6) to give a concentration of 50 µg∙mL^−1^. Twenty microliters (20 µL) of this solution were deposited in a microtiter plate and incubated overnight at 4 °C. The plate was then washed three times using 200 µL of 0.1 M PBS Tween-20 (0.05 *v*/*v*). Two hundred microliters (200 µL) of 1% BSA was then added to block the plates by incubating at 37 °C for 2 h. The plate was then washed and 100 µL of serial dilution of the *Pf*HRP 2 antigen was added to the plate as a 0.01, 0.5, 0.1, 1.0, 10, 50, and 100 µg∙mL^−1^ antigen diluted in PBS (0.01 M). The control wells contained no antigen. The plate was then incubated at 37 °C for 2 h. The plate was washed three times in PBS Tween-20 (0.05 *v*/*v*). Two hundred microliters (200 µL) of *Pf*HRP 2 detection antibody-HRP (HCA 159) were dissolved in 800 µL of PBST-20 (0.05 *v*/*v*) to give a concentration of 20 µg∙mL^−1^, and incubated at 37 °C for 2 h. The plate was washed three times using 200 µL of 0.01 M PBS Tween-20, (0.05 *v*/*v*). One hundred microliters (100 µL) of 3, 3′, 5, 5′ Tetramethylbenzidine were added to the reaction wells, and incubated at room temperature for 15 min in the dark. The reaction was stopped using 50 µL of 1 M H_2_SO_4_ and read at a 450 nm wavelength. The standard curve and linear regression with the limit of detection were obtained in Microsoft Excel. The limit of detection (LOD) was calculated as 3 times the SD of the blank measurement plus the average blank measurement.

### 2.5. Optimisation of Capture and Detection Antibody on the Sensor’s Surface

A 20 µL capture antibody in concentrations of 10, 20, and 30 μg∙mL^−1^ in sodium bicarbonate buffer (0.1 M, pH 9.6) was immobilised by physical adsorption on the gold working electrodes (overnight at 4 °C) in humid conditions. Prior to the immobilisation of the antibodies, the sensors were cured at 120 °C and washed using distilled water. After the immobilisation, the sensors were washed twice using PBST and dried in a gentle flow of nitrogen. The electrode surface was blocked using 100 μL of 1:10 milk in PBS (0.01 M) to reduce non-specific binding and incubated for 1 h at 37 °C. After each incubation step, the surface was washed gently with 100 µL PBST. After washing, 30 µg∙mL^−1^ of *Pf*HRP 2 antigen prepared in 20 μL of PBS was dropped onto the working electrode and incubated for 1 h at 37 °C. The surface was washed with PBST again. The detection antibody HCA 159P was diluted to a working strength of 30 μg∙mL^−1^ in 1:40 milk concentrate and incubated on the sensor for 1 h. The sensors were then washed and assayed by using a volume of 100 μL of the TMB-H_2_O_2_ substrate, dropped on the sensor surface covering all three electrodes. The current was then measured using the potentiostat.

The detection antibody concentration was optimised by repeating the above procedure using the best concentration of capture antibody obtained in the above experiment (20 μL *Pf*HRP 2 capture antibody, 20 μg∙mL^−1^) in sodium bicarbonate buffer (0.1 M, pH 9.6). The capture antibody was immobilised, and the sensor washed and blocked as above. A 30 µg∙mL^−1^ *Pf*HRP 2 antigen was prepared in 1:10 milk PBS, and 10 μL was dropped onto the working electrode. The detection antibody was used at different concentrations of 10, 20, and 30 μg∙mL^−1^ in 1:40 milk PBS. The experiment was repeated as above.

### 2.6. Standard Curve and Limit of Detection

The *Pf*HRP *2* antigen was assayed using 20 μL of capture antibody with a concentration of 20 μg∙mL^−1^ in sodium bicarbonate buffer (0.1 M, pH 9.6) immobilised on the gold working electrode and incubated overnight at 4 ºC. Concentrations of 0, 2, 16, 20, 40, 64, 80, and 100 ng∙mL^−1^ of *Pf*HRP 2 antigen were prepared in 1:10 milk PBS (0.01 M), and 20 μL of the antigen solution was dropped onto the working electrode. The detection antibody-HRP was diluted to a working strength of 20 μg∙mL^−1^ in 1:40 milk PBS. The lowest detection limit of the antigen was determined by 3 times the standard deviation of the blank value plus the average of the blank measurement, and the data were presented using Microsoft Excel.

### 2.7. Signal Amplification Using Gold Nanoparticle

**Scheme 1:**
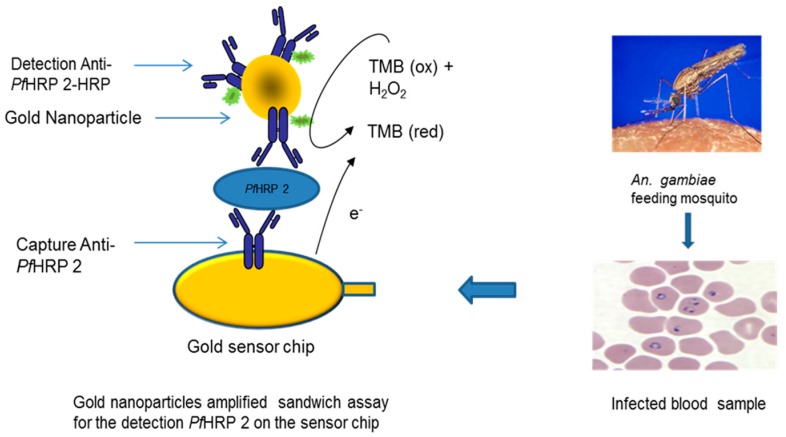
Principle of the developed sensor for malaria detection.

Colloidal gold (40 nm) was employed for its large surface area in an attempt to amplify the sensor’s signal and lower the detection limit of the target protein. The commercial colloidal gold nanoparticles were investigated using different concentrations of blocking buffer for the optimisation of the Au nanoparticles’ (AuNP) conjugation to the reporter protein. A 1000 μL quantity of gold colloid was taken in a 1.5 mL tube, and 0.1 μL of 0.2 M NaOH was added, adjusting the pH to 9.0 [30]. A 100 μL volume of neat 0.1 mg∙mL^−1^ detection antibody (HCA 159P) was added, and the mixture was shaken at room temperature for 1 h. The blocking buffer dilutions of 1:5, 1:10, 1:20, and 1:50 BSA and 1:5 and 1:10 milk in PBS were examined as the blocker after the antibodies’ attachment to the nanoparticles. The tube was then shaken at room temperature for 1 h in the dark and spun at 10,000 rpm for 10 min (4 °C). The supernatant was discarded and the pellet re-suspended in 70 μL PBS (0.01 M) to obtain the stock AuNP—conjugated to the detection antibody—HRP, which was stored at 4 °C. The stock was diluted 1:5 and 1:10 to produce the amplified signal. Another batch of nanoparticles was also prepared in a way similar to the above procedure, but by adding extra horseradish peroxidase enzyme (HRP) after the antibody-HRP attachment. Three microliters (3 μL) of HRP (20 mg∙mL^−1^) were added to the AuNP antibody-HRP solution and incubated for 1 h at room temperature in a shaker. The tube was then spun at 10,000 rpm for 10 min (4 °C) and the supernatant discarded. The sediment was re-suspended in 1 mL distilled water and the procedure continued as used above. The principle of the developed sensor can be seen in Scheme 1.

### 2.8. Human Serum Assay

In order to test the sensor for matrix effect, a commercially available human serum sample was used and spiked with different concentrations of the biomarker. Tests were then conducted using 100% human serum samples following the same procedure as that reported in Section 2.6 and Section 2.7.

## 3. Results and Discussion

### 3.1. Characterisation of the Screen-Printed Electrodes

**Figure 1:**
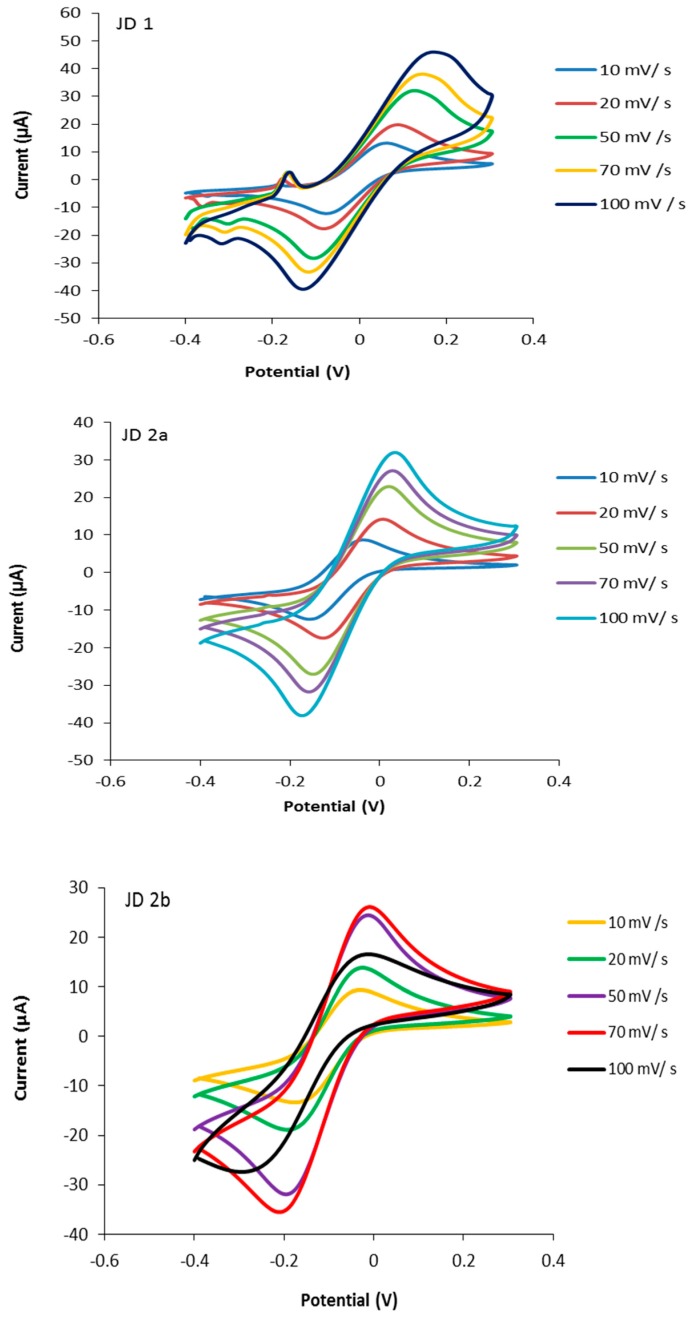
Cyclic voltammogram of the different electrodes at different scan rates using a 1 mM potassium ferricyanide solution in 0.1 M KCl, n = 3.

In order to make a comparison of the 3 electrodes (JD1, JD2a, and JD2b), electrochemical characterisation was conducted using cyclic voltammetry. This was to investigate the performance of the new carbon ink used in the JD2 electrodes, and also to study the effect of the production year on the performance of the sensors. All of the electrodes were stored at room temperature in dark conditions. The experiments were carried out in the presence of potassium ferricyanide, in different concentrations (0.1, 0.5 and 1 mM) and at different scan rates (10, 20, 50, 70 and 100 mV·s^−1^) relative to the on board Ag/AgCl reference electrodes. The cathodic and anodic peak current was used to calculate the active surface area of the electrodes by employing the Randle–Sevcik equation [28,29]. The ideal ΔE value for a reversible redox reaction of potassium ferricyanide is 56 to 59 mV, and the ratio between the cathodic and anodic peak is 1 [31,32]. In practice, however, the difference is typically 100 mV and higher [32]. The use of 1 mM potassium ferricyanide resulted in the best reproducibility of the redox reaction (Data not shown). Figure 1 shows the characterisation of the different sensors using CV with 1 mM potassium ferricyanide.

The results showed that even though JD1 was produced in 2010, it performed well when compared to the JD2 electrodes. It was also noted that the active surface area of the gold working electrode (A_active_ %) in the JD1 was ~10% higher than in the JD2 electrodes (Supplementary information, Table S1). This could be due to physical/chemical changes taking place in the inks/polymer as the electrodes become older. Compared to the JD2 electrodes, JD1 suffered from lower reproducibility. The change in the base carbon ink showed no effect on the sensor’s performance. Both JD2a and JD2b showed comparative data, and further experiments continued using the JD2b electrodes. In order to study the optimal potential for the detection system, the current signals generated from TMB/H_2_O_2_ with the HRP-antibody conjugate were analysed using chronoamperometry. The ratio of the signal current to the background current using step amperometry (−400 mV to +600 mV) of 4 mM TMB and 0.06% H_2_O_2_ with and without the addition of the detection antibody-HRP in a pH 5.0 citrate buffer, 0.1 M KCl, was calculated. The results showed that the best potential in this system is −0.2 V using JD2 electrodes, and therefore this was selected for future immunosensor developments (Supplementary information, Figure S1).

### 3.2. SEM, ESEM and AFM of Bare SPGE

**Figure 2:**
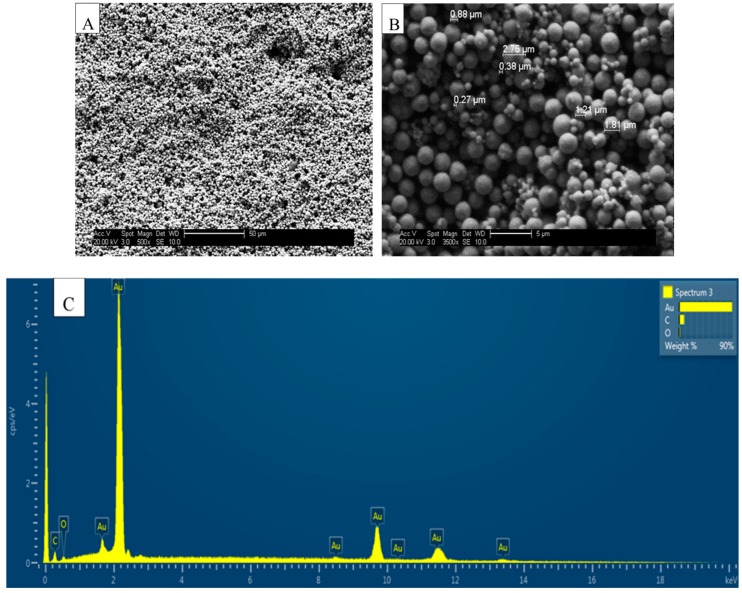
SEM of a JD2b bare electrode, (**A**) at 500×, (**B**) at 3500×, (**C**) ESEM surface analysis of JD2b.

The working electrode of the screen-printed gold sensor was characterised using scanning electron microscopy (SEM) and environmental scanning electron microscopy (ESEM), which show the composition of the gold electrode’s surface (Figure 2). The SEM scans showed pinholes in the surface structure, which were formed as a result of the printing process with a rough granular surface. The ESEM analysis (Figure 2C) gives a small figure insert with the average of three spectra’s data and indicates a high percentage of the gold ink (~89.2%) used to produce the sensors with carbon (~9.11) and oxygen (~1.69).

**Figure 3:**
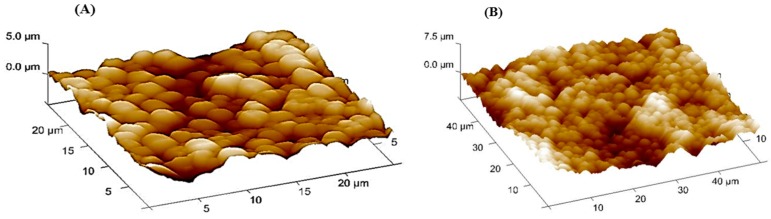
Three-dimensional (3D) surface topography of a bare screen-printed gold electrode (SPGE) using AFM at (**A**) 25 mu and (**B**) 50 mu.

The surface roughness of the gold electrode was visualised using atomic force microscopy (AFM) at 25 and 50 mu (Figure 3). This shows the screen-printed gold working electrode’s topography, similar to the SEM scans.

### 3.3. Development of the Immunoassay

First, the *Pf*HRP 2 assay was developed using the microtiter plate by a direct and a sandwich assay format in order to investigate the suitability of the reagents for the detection of *Pf*HRP 2, before moving the assay to the sensor’s surface. The direct assay was conducted by immobilising the antigen by physical adsorption to the plate, and detecting it using the detection antibody-enzyme conjugate. The sandwich assay used the capture antibody immobilised on the plate surface, and the antigen was added in solution using different concentrations. The detection antibody-enzyme was then added to complete the assay. The results for both assays are shown in the Supplementary Information Figures S2 and S3. The LOD was calculated as 0.56 µg∙mL^−1^ for the direct ELISA and 0.89 µg∙mL^−1^ for the sandwich ELISA. The results reveal that the HuCAL sandwich pair recognises and interacts with the malaria protein, and can be used in the development of an immunosensor. No further development of the assay or optimisation was conducted on the ELISA assay, since the aim of the work was to focus on the sensor’s development.

### 3.4. Development of PfHRP 2 Immunosensor

**Figure 4:**
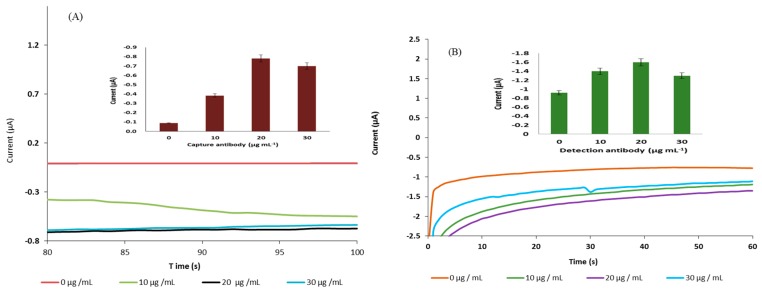
Chronoamperometric response of (**A**) different concentrations of capture antibody on the sensor’s surface in a sandwich assay format; (**B**) different concentrations of detection-horseradish peroxidase (HRP) antibody in a sandwich assay format, n = 3.

Capture antibody optimisation was conducted using a sandwich ELISA format. Concentrations of 10, 20, and 30 µg∙mL^−1^ were added to the sensor’s surface (20 µL in sodium bicarbonate buffer, 0.1 M, pH 9.6) to attach to the sensor using physical adsorption (overnight at 4 °C). The electrodes were then blocked using 100 µL, 1:10 milk in PBS (0.01 M) for 1 h at 37 °C, and then washed gently using PBST buffer. The *Pf*HRP 2 antigen was then dropped onto the sensor’s surface (20 µL, 30 µg∙mL^−1^) and incubated for 1 h (37 °C), washed, and then the detection antibody-enzyme (20 µL, 30 µg∙mL^−1^) was added and incubated (1 h, 37 °C). The assay was then followed by adding the TMB-H_2_O_2_ substrate, and the signal was recorded using a −200 mV potential. TMB-H_2_O_2_ was chosen as the enzyme substrate for the enzyme label horseradish peroxidase (HRP)’s activity determination [33]. Furthermore, TMB has superior detection properties than other systems [34,35,36]. Figure 4A shows that the best concentration was found to be 20 µg∙mL^−1^ for the capture antibody. The response increased linearly against antibody concentration up to about the 20 µg∙mL^−1^ concentration level, and after this point the response was lower, which indicates the saturation of the sensor’s surface. A 20 µg∙mL^-1^ concentration of anti *Pf*HRP 2 antibody was chosen as the optimum concentration for the capture antibody, since it was the best compromise between the response and the cost of the antibody.

The detection antibody concentration was then optimised using 20 µg·mL^−1^ capture antibody immobilised on the sensor’s surface. The detection antibody-enzyme conjugate was tested at 10, 20, and 30 μg∙mL^−1^ in 1:40 milk PBS and 30 µg∙mL^−1^ antigen. The procedure followed was similar to that listed above. The results are shown in Figure 4B, with the highest signal recorded at 20 µg∙mL^−1^ also for the detection antibody-HRP concentration.

**Figure 5:**
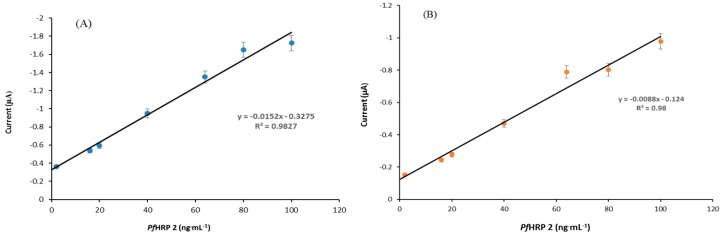
Linear regression analysis of the chronoamperometric response of *Pf*HRP 2 detection in buffer (PBS, 0.01M, pH 7.4, spiked with 1–100 ng∙mL^−1^ *Pf*HRP 2) (**A**) and 100% serum samples (spiked with 1–100 ng∙mL^−1^ *Pf*HRP 2) (**B**) on a JD2 sensor. Measurements were conducted at −0.2 V. Correlation coefficient and R^2^ value of 0.9827 for the buffer matrix and 0.98 for the serum matrix (n = 3).

The immunosensor was then developed for the detection and quantification of *Pf*HRP 2. The optimal capture (20 µg∙mL^−1^) and detection antibody (20 µg∙mL^−1^) concentrations were used with the JD2 electrodes to conduct a calibration curve. Different *Pf*HRP 2 antigen concentrations (0, 2, 16, 20, 64, 80, and 100 ng∙mL^−1^) prepared in 1:10 milk/PBS (0.01M) as dilution buffer, and then a set of experiments was conducted in spiked 100% commercial human serum samples. The assay was run in triplicate for all of the measurements. The blank contained no antigen for the buffer and the serum experiments. The results of both assays are shown in Figure 5. The results agree with the range used by [37] for the detection of *PfHRP 2*. The effect of tandem repeats in the structure of the protein make the antigen easy to detect; however, the concentration of the analyte is influenced by the matrix.

From the data shown in Figure 5A, the limit of detection (LOD) for the buffer samples was calculated as 2.14 ng∙mL^−1^, and for the spiked human serum samples (Figure 5B) the LOD was 2.95 ng∙mL^−1^. These results show that both assays give a similar detection limit, but with lower readings achieved for the serum samples. This is very encouraging and indicates that the sensors are able to perform well using 100% commercial human serum samples. The difference in matrix affects the rate of electron exchange, which occurs when the analyte is oxidized when a potential difference is applied. The lower readings can be due to small proteins in the samples attaching to the sensor’s surface and affecting the electron transfer. The sensitivity and reproducibility of the sensors in this assay are shown to be adequate for the detection of the malaria biomarker, since a blood level of ~ 9.45 ng∙mL^−1^ has been reported to be *Plasmodium* sp. specific and malaria positive [38,39,40].

**Figure 6:**
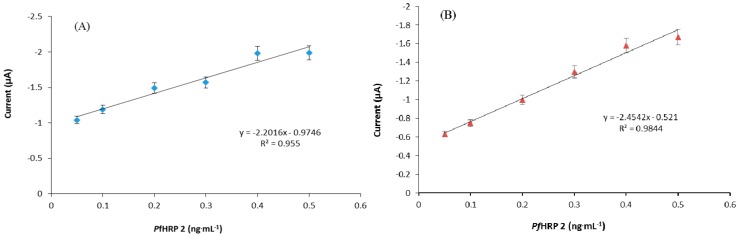
Linear regression of the chronoamperometric response of *Pf*HRP 2 detection in buffer (PBS, 0.01M, pH 7.4, spiked with 0.05–0.5 ng∙mL^−1^ *Pf*HRP 2) (**A**) and 100 % spiked serum samples (Commercial human serum samples spiked with 0.05–0.5 ng∙mL^−1^ *Pf*HRP 2) (**B**) on JD2 sensors using gold nanoparticles conjugated to the detection antibody-HRP. Measurements were conducted at −0.2 V. Correlation coefficient and R^2^ value of 0.955 for the buffer matrix and 0.9844 for the serum (n = 3).

To investigate if we can improve the detection limit further, the detection antibody-HRP was attached to gold nanoparticles (40 nm) and used in the sandwich assay. Several optimisation experiments were conducted to achieve the best results before a calibration curve was repeated with serial dilution of the antigen in buffer first, and then the experiments were repeated in 100% commercial human serum samples. The full procedures are listed in Section 2.7. Figure 6 shows the linear regression of the results achieved using the amplified assay with the gold nanoparticles conjugated detection antibody-HRP alone, without the addition of free enzymes to attach to the gold nanoparticles.

From the above data, an LOD of 36 pg∙mL^−1^ was obtained in the amplified buffer samples with an R^2^ value of 0.955 (Figure 6A). An LOD of 40 pg∙mL^−1^ was also obtained in the amplified 100% serum samples (Figure 6B). The AuNP results gave excellent sensitivity and limit of detection without the use of additional free enzymes (horseradish peroxidase) to load the particles. The best AuNP conjugate stock dilution used was 1:10 milk in PBS, and also blocking with milk proteins. The serum proteins in the samples in this assay also showed a similar trend to the non-amplified assay, in that similar responses where achieved to the buffer samples. Table 1 shows some of the different biosensor technology for detecting *P*fHRP 2, and their detection limits. These are comparable to the limits achieved using our sensor. Our work showed that a proof of concept sensor was developed and this can achieve a much lower detection limit than is required for malaria’s positive detection in blood samples. Further work will be done to examine the sensor in patient serum samples to confirm the results achieved in this work.

## 4. Conclusions

An immunosensor has been successfully developed using a sandwich ELISA assay on JD2 gold screen-printed electrodes. Milk concentrate was used as the blocking protein, as it reduced non-specific binding on the electrode surface. With both malaria antigen and antibodies being very expensive, care had to be taken in designing the experiments to achieve optimised results. An ELISA test was first developed to check the affinity of both antibodies toward the antigen *Pf*HRP 2. An immunosensor was then developed and optimised with electrochemical measurements that produced a 2.14 ng∙mL^−1^ detection limit for the buffer samples, which is better than the ELISA assay developed in this work. Spiked 100% serum samples also achieved a very good LOD of 2.95 ng∙mL^−1^. An amplified signal is also achievable using the sensor with AuNPs conjugated to the detection antibody-enzyme. Signal amplification using gold nanoparticles gave an LOD of 36 pg∙mL^−1^, while the serum assay gave an LOD of 40 pg∙mL^−1^. The developed immunosensor offers a highly sensitive, portable, and low cost method of detecting *Plasmodium falciparum* histidine-rich protein 2. Future experiments will look at real samples analysis using patient serum samples.

## Figures and Tables

**Table 1:** Different biosensor technology for detecting *P*fHRP 2, and their detection limits.

Analyte	Assay Principle	Range	Detection Limit	References
*Pf*HRP 2	SPR	-	5.6 pg∙mL^−1^	[25]
*Pf*HRP 2	Carbon SPE modified with MWCN and Au/MWCN	-	8 ng∙mL^−1^	[37]
*Pf*HRP 2	Carbon SPE modified with AuNPs/Al_2_O_3_sol–gel	-	-	[41]
*Pf*HRP 2	Graphite–epoxy composite magneto electrodes	-	0.36 ng∙mL^−1^	[42]
*Pf*HRP 2	Polydimethylsiloxane microfluidic chips	-	16 ng∙mL^−1^	[43]
*Pf*HRP 2	Carbon nanofiber forest grown on glass microballons	0.01–10 ng∙mL^−1^	0.025 ng∙mL^−1^	[44]
*Pf*HRP 2	Mercaptopropylphosphonic acid functionalized copper doped zinc oxide nanofibers	10 ag∙mL^−1^–10 µg∙mL^−1^	6.8 ag∙mL^−1^	[45]
*Pf*HRP 2	Low electrocatalytic indium tin oxide (ITO) on glass electrodes; APTES-glutaraldehyde modified	1 pg∙mL^−1^–100 ng∙mL^−1^	2.2 pg∙mL^−1^	[46]

## Supplementary Materials

Figure S1: Optimum potential determination by step potential of TMB/H_2_O_2_ system with antibody-HRP on JD2 electrodes. The results shown are after subtracting the signal with no enzyme, Figure S2: (**A**) Standard curve of absorbance versus antigen concentration in a direct ELISA assay, (**B**) linear regression with correlation coefficient and R^2^ value of 0.9612, limit of detection is 0.56 µg∙mL^−1^, Figure S3: (**A**) Standard curve of absorbance versus antigen concentration in a Sandwich ELISA assay, (**B**) linear regression with correlation coefficient and R^2^ value of 0.9755. Limit of detection is 0.89 µg∙mL^−1^, Table S1: Overview of cyclic voltammetric analyses of the three electrodes, JD1, JD2a and JD2b at 20 mV·s^−1^, using 1 mM potassium ferricyanide solution in 0.1 M KCl, n = 5.
