# Supplementary material for: Development of an Immunosensor for PfHRP 2 as a Biomarker for Malaria Detection
Source: Biosensors (Basel). 2017 Jul 18;7(3):28. doi: 10.3390/bios7030028 (PMC5618034; doi:10.3390/bios7030028)
Supplement: Supplementary file 1 [file biosensors-07-00028-s001.pdf]

Development of an Immunosensor for *Pf*HRP 2 as a Biomarker for Malaria Detection

Aver Hemben [1], Jon Ashley [1, 2] and Ibtisam E. Tothill [1]*

[1] Surface Engineering and Nanotechnology Institute, Cranfield University, Cranfield, Bedfordshire MK43 0AL, England, UK

[2] Department of Micro- and Nanotechnology, Technical University of Denmark, Technical University of Denmark, Produktionstorvet, 2800 Kgs. Lyngby, Denmark

----

*Corresponding author

I.E. Tothill

Tel: +44 (0) 7500766487

E-mail: i.tothill@cranfield.ac.uk

Table S1: Overview of cyclic voltammetric analyses of the three electrodes, JD1, JD2a and JD2b at 20 mV s^{-1}, using 1 mM potassium ferricyanide solution in 0.1 M KCl, n= 5.

	**JD1**	**JD2a**	**JD2b**
**I_{PA} (µA)** [a]	19.88 ± 0.18	14.41 ± 0.32	13.70 ± 1.12
**E_{PA} (V)** [b]	0.09 ± 0.01	0.004 ± 0.001	-0.03 ± 0.01
**I_{PC} (µA)**	-24.99 ± 0.02	-17.43 ± 0.01	-18.93 ± 0.67
**E_{PC} (v)**	-0.079 ± 0.11	-0.13 ± 0.01	-0.20 ± 0.02
**ΔE (V)** [c]	0.17	0.13	0.17
**I_{PA} / I_{PC}** [d]	-1.14	-0.83	-0.72
**A_{active}**	0.18	0.15	0.16
**Aactive (%)** [e]	78.9	66.8	69.6

[a] $I_{P\ C/A}$ = Cathodic / anodic peak current
[b] $E_{P\ C/A}$ = Potential applied at the cathodic / anodic peak
[c] Peak distance ΔE = E_{PA} – E_{PC}
[d] Ratio between the cathodic and anodic peak current
[e] Ratio between the active area calculated by the Randles – Sevick equation and the geometric surface.

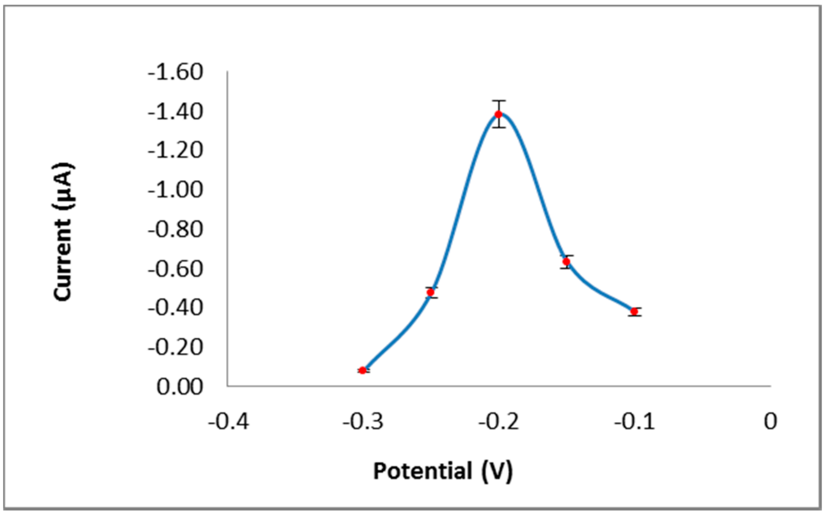

Fig. S1. Optimum potential determination by step potential of TMB / H_2O_2 system with antibody-HRP on JD2 electrodes. The results shown are after subtracting the signal with no enzyme.

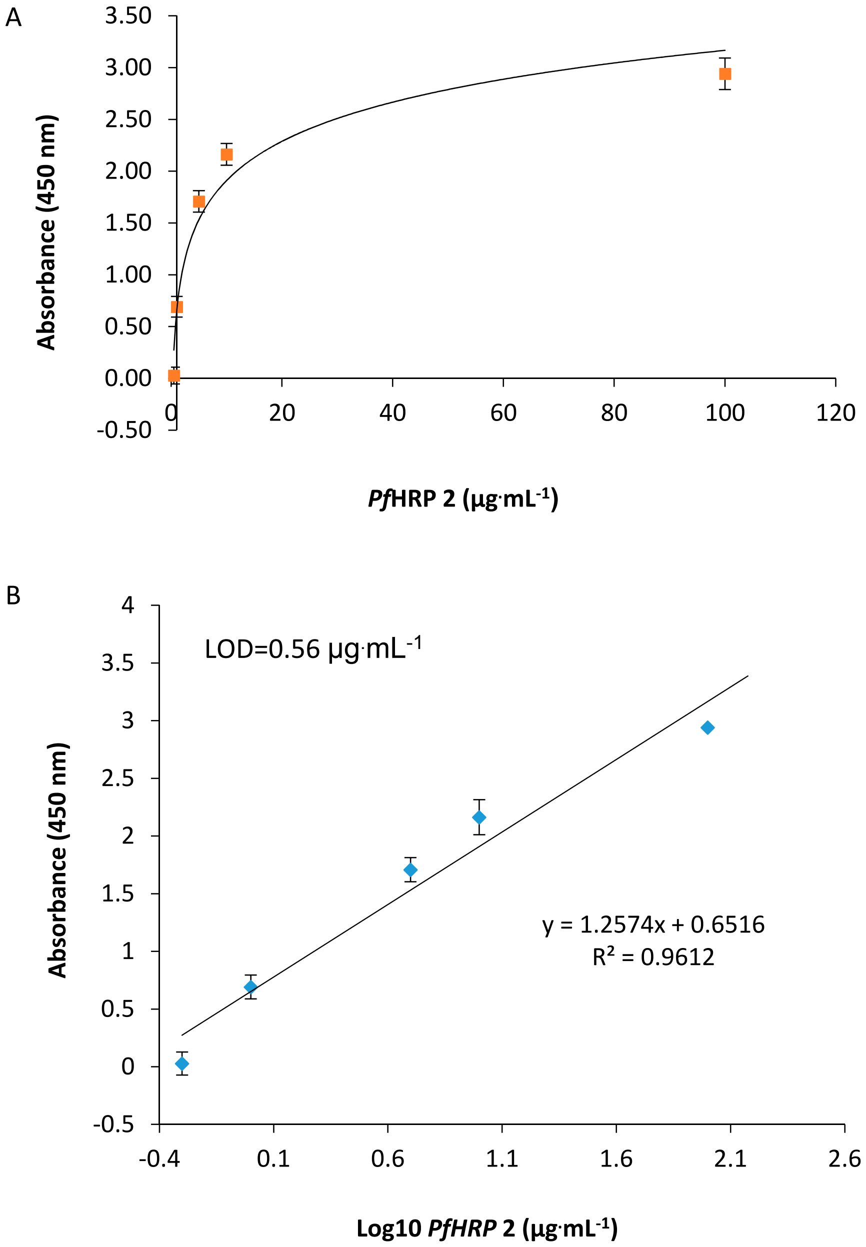

Fig. S2. (a) Standard curve of absorbance versus antigen concentration in a direct ELISA assay, (b) linear regression with correlation coefficient and R^2 value of 0.9612, limit of detection is 0.56 µg· mL^{-1}.

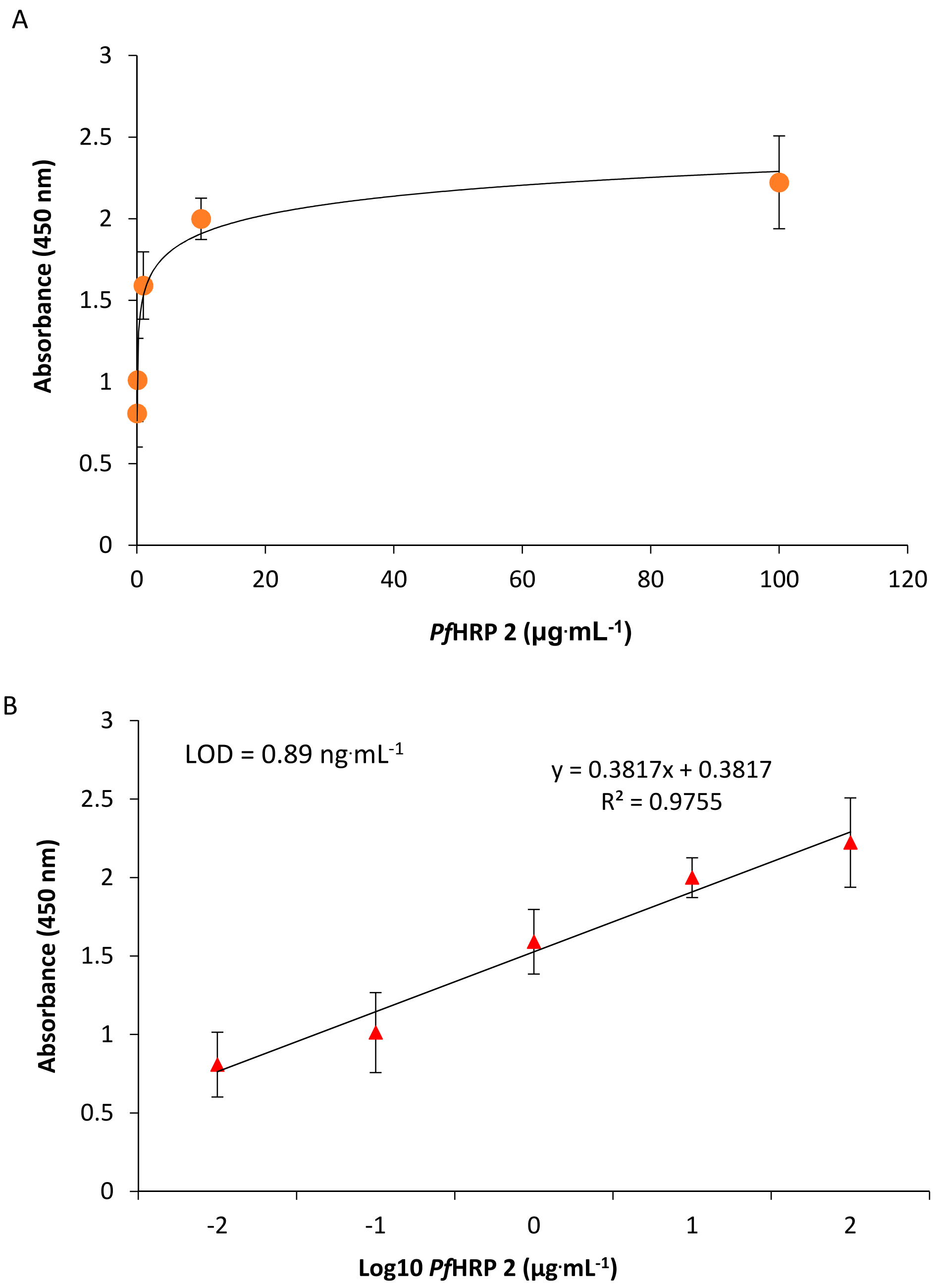

Fig. S3. (a) Standard curve of absorbance versus antigen concentration in a Sandwich ELISA assay, (b) linear regression with correlation coefficient and R^2 value of 0.9755. Limit of detection is 0.89 µg mL^{-1}.
